# Strain Dependence of Metal Anode Surface Properties

**DOI:** 10.1002/cssc.202000709

**Published:** 2020-05-27

**Authors:** Daniel Stottmeister, Axel Groß

**Affiliations:** ^1^ Institute of Theoretical Chemistry Ulm University 89069 Ulm Germany; ^2^ Helmholtz Institute Ulm (HIU) Electrochemical Energy Storage Helmholtzstr. 11 89069 Ulm Germany

**Keywords:** calcium, density functional theory, energy transfer, lithium, potassium

## Abstract

Dendrite growth poses a significant problem in the design of modern batteries as it can lead to capacity loss and short‐circuiting. Recently, it has been proposed that self‐diffusion barriers might be used as a descriptor for the occurrence of dendrite growth in batteries. As surface strain effects can modify dendritic growth, we present first‐principles DFT calculations of the dependence of metal self‐diffusion barriers on applied surface strain for a number of metals that are used as charge carriers in batteries. Overall, we find a rather small strain dependence of the barriers. We mainly attribute this to cancellation effects in the strain dependence of the initial and the transition states in diffusion.

## Graphical abstract

**To strain or not to strain**: We calculate the strain dependence of metal anode surface properties from first principles to explore whether surface stress can be related to dendrite growth in metal‐ion batteries on the atomistic level. We only find a weak dependence of these properties on the applied strain, which suggests that strain does not influence dendrite growth on the atomistic scale, but rather dendrite‐related morphologies on longer‐length scales.

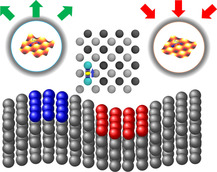

## Introduction

Since their introduction in 1991, Li‐ion batteries have become the dominant energy storage technology for portable devices and have seen various improvements over the years, which has allowed them to approach their theoretical energy density.^1^ With the worldwide increase in required energy storage capacities, Li‐based battery systems now face new problems related to the acquisition of sufficient amounts of Li. Lithium is relatively scarce within the earth's crust and concentrated in remote locations, which leads to increased competition over the limited resources.^2^ Difficulties in Li mining combined with an increasing worldwide demand for Li has already led to a substantial rise in the cost of Li.^3^ Apart from economic consequences, Li mining is problematic from an ecological point of view, as it consumes vast amounts of water in, generally, water‐starved regions.^4^ To tackle these growing problems within the field of electrochemical energy storage, new battery systems that do not rely on Li have to be developed. Promising candidates for such post‐Li batteries include other alkali metals such as Na^5^ or K^6^ and multivalent materials such as Ca,^7^ Al,^8^ Mg,^9^ and Zn.^10^

Still, much like their predecessor, post‐Li batteries suffer from a variety of technical problems, arguably the worst of which is the lack of long‐term stability of the electrodes.^11^ For many materials, the formation of so‐called dendrites is a significant contributor to capacity loss because of the creation of dead metal, but their growth also creates safety risks that stem from internal short‐circuiting.^12^ Extensive studies on dendritic growth have been performed for Li battery systems,^13^, ^14^ and several possible solutions for the suppression of dendrites have been proposed.^15^, ^16^ One of these solutions includes the introduction of electrolyte additives, such as Cs^+^, HF, and LiNO_3_, however, unfortunately these electrolyte additives are usually consumed in side reactions.^17^, ^18^, ^19^, ^20^ Another approach includes the implantation of a presynthesized solid–electrolyte interface (SEI) on the electrode.^21^

It is important that any modifications of the SEI do not compromise the desired properties of the SEI. In particular, a high ion conductivity and, consequently, a high number of diffusion pathways are necessary to ensure a uniform surface growth distribution.^21^ One aspect that has recently gained attention is the role of strain effects in battery operation.^22^ Although the influence of strain rates on surface properties is not a new concept and is known widely within the field of catalysis,^23^, ^24^, ^25^ its impact on battery properties and even its potential for application within batteries is not yet fully understood. The presence of compressive stress during plating has been demonstrated, and a stress‐driven dendrite growth mechanism has been proposed for Li.^26^, ^27^ Recently, the formation of Li whiskers as a direct consequence of applied stress was observed by using in situ environmental transmission electron microscopy,^28^ which further demonstrates the importance of strain effects for battery development. However, a current continuum modeling study could only find a stress‐induced suppression of dendrite growth if stress heterogeneities on a length scale larger than that of single dendrites is taken into account.^29^

Previously, we suggested that the height of self‐diffusion barriers could be used as a descriptor for dendrite growth in batteries.^12^, ^30^ In an attempt to refine the model and include the electrochemical environment, we have recently also considered the impact of electric fields on self‐diffusion barriers.^31^ We are now further extending this model by studying the influence of strain on the self‐diffusion barriers to provide a better understanding of the underlying mechanics that govern dendrite growth. Interestingly, only a few studies so far have been devoted to strain effects in surface diffusion.^32^ As far as the first‐principles treatment of these strain effects are concerned, there has been one seminal study on the strain dependence of surface diffusion in the diffusion of Ag atoms on Ag(1 1 1).^33^ This study showed a linear strain dependence of the Ag self‐diffusion barrier, which is, however, relatively small, the barriers change by only approximately 20 meV if the lattice constant is changed by 5 %. Here we present periodic DFT calculations performed to address the influence of strain on the surface properties of Li and post‐Li metal anode systems and we will discuss the consequences of our findings for the understanding of dendrite growth in batteries.

## Computational Details

We performed the calculations in this work by applying DFT using the plane‐wave‐based Vienna ab initio simulation package (VASP).^34^ The exchange–correlation was calculated by using the exchange–correlation functional according to Perdew, Burke, and Ernzerhof (PBE)^35^ within the generalized gradient approximation (GGA). The electron–core interactions were described by the projector augmented wave (PAW) method.^36^, ^37^ The cutoff values were chosen for each element to reproduce known bulk lattice constants and cohesive energies. The metal surfaces were modeled by using a seven‐layer slab with a 4×4 geometry and a vacuum region of *>*20 Å. A *Γ*‐centered 5×5×1 *k*‐point grid was used to calculate the energies. The electronic self‐consistent‐field (SCF) scheme was converged up to 10^−5^ eV by using the Methfessel–Paxton smearing scheme^38^ with a width of 0.2 eV, and the ionic geometry was converged to energetic differences below 10^−4^ eV. In our calculations, we considered a maximum strain of ±3 %, as larger strains in metal films are typically released by the formation of dislocation networks.^39^

## Results and Discussion

### Bulk properties

We will first present the results for the different metal systems without any applied strain and then discuss the strain effects for various properties of the metal surfaces. The metal cohesive energies *E*_coh_ were determined by subtracting the energy of the isolated atom in a vacuum *E*_vac_ from the corresponding bulk energy *E*_bulk_ per atom [Eq. (1)]:

(1) $$E{}_{\mathrm{coh}}=E{}_{\mathrm{bulk}}-E{}_{\mathrm{vac}}$$

The lattice constants were derived from the minimum of the cohesive energy. Our calculated results are compared with experimental data in Table 1.^40^ Notably, the considered metals crystallize in different equilibrium configurations at room temperature: Li, Na, and K as body‐centered cubic (bcc), Mg and Zn as hexagonal close packed (hcp), and Al and Ca as face‐centered cubic (fcc) structures. We find that our calculated lattice constants are typically slightly underestimated, whereas the cohesive energies are in rather good agreement with the experimental values. The cohesive energy of Zn was, however, not very well represented within the parameters employed in this work. Notably, an increase in the used cutoff value or the *k*‐point density did not improve the cohesive energy of Zn. The underestimation of the Zn cohesive energy in PBE‐DFT calculations has been found before.^41^ To avoid different setups for the various metals, we did not attempt to find a functional better suited for Zn, however, because of this comparatively poor representation of the Zn cohesive energy within this work, the results for Zn should be viewed with caution.

**Table 1:** Values for the calculated and the literature lattice constants and cohesive energies *E*_coh_ for all investigated systems.

Metal	*a*_calc_	*a*_literature_ 40	*c*_calc_	*c*_literature_ 40	*E*_coh,calc_	*E*_coh,literature_ 40
	[Å]	[Å]	[Å]	[Å]	[eV]	[eV]
Li	3.441	3.510	–	–	−1.61	−1.63
Na	4.192	4.291	–	–	−1.09	−1.11
K	5.323	5.328	–	–	−0.88	−0.93
Mg	3.219	3.209	5.123	5.211	−1.50	−1.51
Ca	5.527	5.588	–	–	−1.91	−1.84
Zn	2.611	2.665	5.094	4.947	−1.10	−1.35
Al	4.040	4.050	–	–	−3.40	−3.39

### Surface energy

The surface energy is a measure of the energy cost to create a particular surface and can be used to estimate the likely surface terminations of a given metal. The surface energy *E*_surf_ is defined as the energy per area required to form a specific surface from a bulk structure. Here *E*_surf_ was calculated by employing two approaches. For a symmetric slab in which the topmost layers on both sides of the slab are relaxed, the surface energy is given by Equation (2):

(2) Esymsurf=Esymrelaxed-Natom×Ebulk2A

in which Esymrelaxed represents the energy of the relaxed surface system, *N*_atom_ is the number of atoms per super cell, and *A* is the surface area of the supercell. These symmetric slab calculations usually require relatively thick slabs that lead to a larger computational effort. However, surface energies can also be derived for thinner asymmetric slabs in which only one side of the slab is relaxed and the other side is kept at its ideal bulk positions according to Equation (3):^42^

(3) Easymsurf=Easymrelaxed-Estatic-Natoms×Ebulk2A

in which Easymrelaxed represents the energy of the slab with one relaxed surface and *E*_static_ is the energy of the static slab in the ideal bulk termination without any relaxation. We compared the surface energies obtained from both approaches to check the reliability of the results, as any significant disparity between the results would indicate an insufficiently large slab size. For all considered metals we calculated the surface energies of the most stable surface terminations with a significant area fraction within the Wulff construction.^43^

Both the symmetric and the asymmetric surface slabs were calculated by using seven‐layer slabs with either the inner three layers or the bottom five layers kept at the ideal bulk positions. Both approaches yield very similar results, which indicates that the calculated surface energies are converged with respect to the slab thickness. We list the results for the asymmetric calculations in Table 2 as they have a larger bulk area. We explicitly compare our results with those of a previous study^12^ and find, in general, a good agreement. The same is true if we compare them with results from another source,^43^ except for the case of the Mg(1 0 1 0) surface.

**Table 2:** Calculated surface energies and self‐diffusion barriers compared to the results of previous computational studies, if available.

Metal	*E*_surf_ [J m^−2^]	*E*_surf‐lit_ [J m^−2^]	*E*_diff_ [eV]	*E*_diff‐lit_ [eV]
Li(1 0 0)	0.46	0.46^43^	0.13	0.14^12^
Li(1 1 0)	0.49	0.50^43^	0.01	0.02^12^
Li(1 1 1)	0.54	0.54^43^	0.40	0.41^12^
Na(1 0 0)	0.24	0.22^43^	0.15	0.16^12^
Na(1 1 0)	0.23	0.22^43^	0.04	0.04^12^
Na(1 1 1)	0.26	0.25^43^	0.27	–
K(1 0 0)	0.12	0.12^43^	0.11	–
K(1 1 0)	0.10	0.11^43^	0.02	–
K(1 1 1)	0.13	0.13^43^	0.22	–
Ca(1 0 0)	0.44	0.46^43^	0.35	–
Ca(1 1 1)	0.45	0.46^43^	0.01	–
Al(1 0 0)	0.90	0.91^43^	0.53	–
Al(1 1 1)	0.79	0.77^43^	0.05	0.05^12^
Mg(0 0 0 1)	0.52	0.51^43^	0.02	0.02^12^
Mg(1 0 1 ¯0)	0.86	0.60^43^	0.28	–
Zn(0 0 0 1)	0.36	0.33^43^	0.01	0.02^12^
Zn(1 0 1 ¯0)	0.53	0.53^43^	0.27	–

To study strain effects, we changed the lattice constants within the range of −3 to +3 %. We did not consider possible phase transitions between different lattice structures induced by strain, as they can occur for Li and Na under high‐pressure conditions.^44^, ^45^ If we look at the surface energy of a strained system, the reference value used to calculate the surface energy for the different surfaces is of importance. If the surface energy was calculated using the strained lattice as a reference, the surface energies for all strained systems would be lower than those in the nonstrained system because of the energetically less stable reference values. This would not be a “wrong” result as it is quite sensible for an energetically less stable bulk system to require less energy to form a surface compared to the optimized bulk system. Within this work, however, a different reference scheme was used in which the chosen reference value was the optimal bulk energy per atom. This is both advantageous, as well as flawed. The advantage is that it allows for a direct comparison of the stability of the surface with the nonstrained surface, whereas the disadvantage is the dependence of the result on the slab thickness (as every nonrelaxed atom in the slab adds to the surface energy). A mixed reference scheme, in which the bulk atom energies would be referenced with the strained values, whereas for the surface atoms, the reference values would be the optimal lattice ones, was considered to compensate the scaling problem of the optimal lattice reference. However, this approach would ignore the energy cost required to strain the underlying slab. It was decided to use the optimal reference scheme that includes the energetic cost of distorting the underlying bulk region of the slab, which allows for a more realistic look at the stability of each strained surface, even though this energy might not strictly fit the definition of the surface energy anymore.

**Figure 1:**
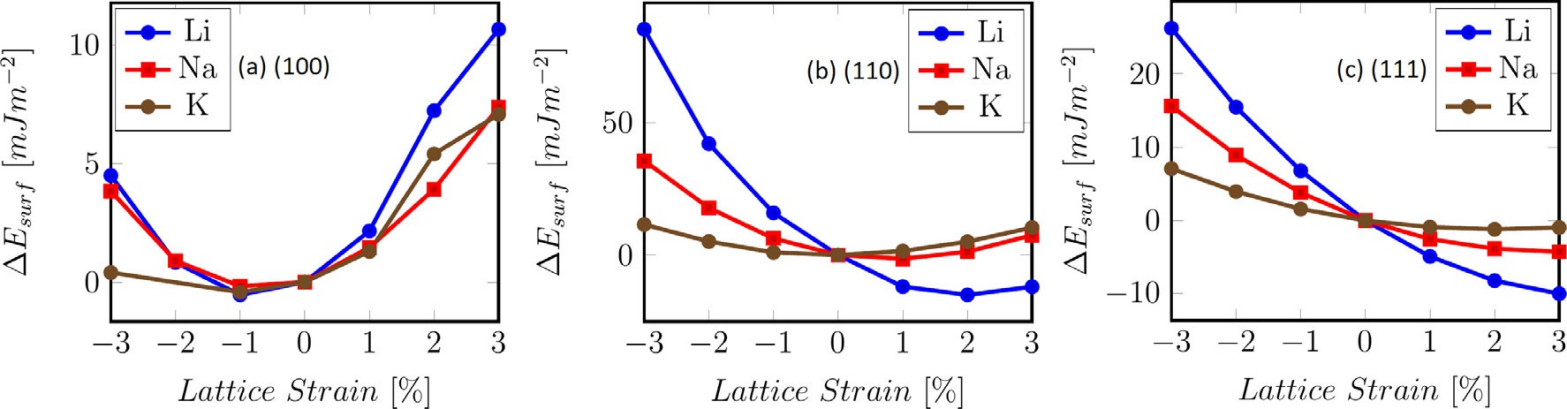
Surface energy with respect to the lattice distortion for the bcc metals Li, Na, and K using the optimal lattice reference (see text).

If we compare the resulting data for the alkali metals shown in Figure 1, several similarities could be observed. The (1 0 0) surface (Figure 1 a) is the most stable under a small compression of −1 % for all three elements Li, Na, and K. In contrast, all (1 1 1) surfaces (Figure 1 c) are more stable under expansion, whereas for the (1 1 0) surfaces (Figure 1 b), only Li and Na show this behavior. A further trend among the surface terminations can be observed; the variation in the surface energy is always the highest for the (1 1 0) surfaces, whereas the (1 0 0) surfaces exhibit the smallest variation. Overall, the change in surface energies was found to be rather minor (*<*0.05 J m^−2^) with the exception of the Li(1 1 0) surface (*>*0.08 J m^−2^). Interestingly, the most densely packed (1 1 1) surfaces and the open (1 1 0) surfaces exhibit the same trend in the surface energies as a function of lattice strain, whereas the (1 0 0) surfaces, which are intermediate with respect to surface roughness, show the opposite trend. Hence there is no simple explanation for the observed trends.

The direct comparison between the different alkali metals (Figure 1) shows a clear hierarchy for the change in surface energy, in which Li always yields the most significant change, whereas K is the least affected. This correlates with the cohesive energies of the corresponding elements. Also, the more densely packed (1 1 1) surface shows a clear preference for a lattice expansion for all three tested alkali metals, whereas the opposite was true for the less densely packed (1 0 0) surface. With respect to the metals not depicted in Figure 1, the change in the surface energy for calcium shows that the (1 1 1) surface is more stable under expansion, whereas the (1 0 0) surface is the most stable under nonstrained conditions. For Al, the nonstrained conditions are favorable for the (1 0 0) and (1 1 1) surface cuts. The energetic changes for Al and Ca are bigger than the changes for the alkali metals by almost one order of magnitude, with the exception of the Li(1 1 0) surface. For the hcp metals, both the Mg(1 0 1 ¯0) and the Zn(1 0 1 ¯0) surfaces are more stable under nonstrained conditions, whereas the Mg(0 0 0 1) surface favors a slight compression and the Zn(0 0 0 1) surface a slight expansion. Mg has energetic changes comparable to those of Ca and Al, whereas Zn shows the most substantial variation with a change of up to 0.8 J m^−2^.

### Adsorption energy

We calculated the metal adsorption energies for the most stable adsorption sites for all considered surfaces according to Equation (4):

(4) $$E{}_{\mathrm{ads}}=E{}_{\mathrm{atom}/\mathrm{slab}}-E{}_{\mathrm{slab}}-E{}_{\mathrm{vac}}$$

in which *E*_atom*/*slab_ is the energy of the slab with one adsorbed atom per surface unit cell, *E*_slab_ is the energy of the clean slab, and *E*_vac_ corresponds to the total energy of the isolated metal atom. All adsorption calculations were performed under the same conditions using two relaxed surface layers.

**Figure 2:**
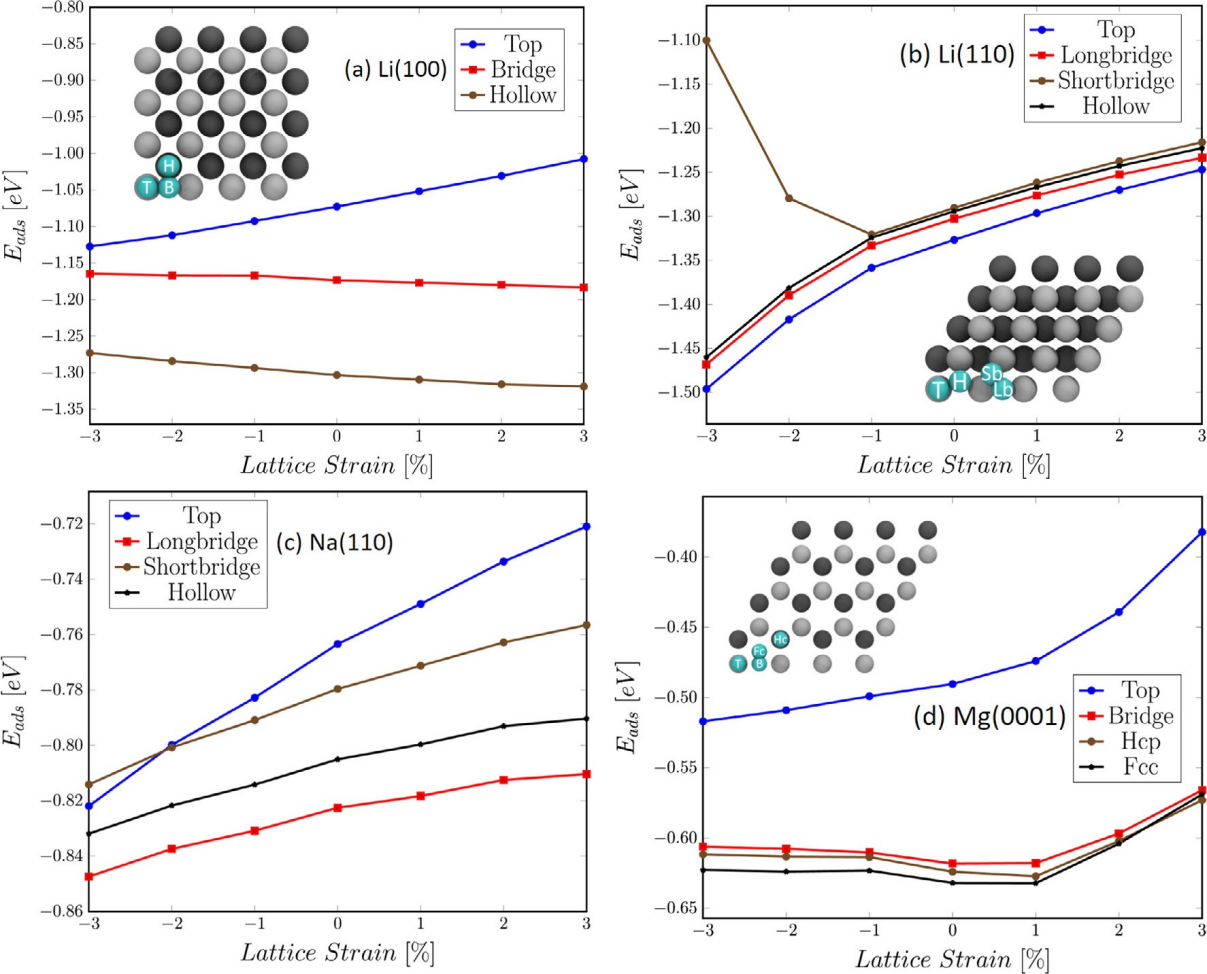
Adsorption energies as a function of lattice strain.

To illustrate the observed trends in the metal adsorption energies, the adsorption energies on Li(1 0 0), Li(1 1 0), Na(1 1 0), and Mg(0 0 0 1) are plotted as a function of lattice strain in Figure 2. We will first describe the observed trends. On Li(1 0 0) (Figure 2 a), at the top position Li adsorption becomes weaker with the increasing strain, in qualitative agreement with the three other considered surfaces, whereas the opposite is true at the hollow site and the bridge site of Li(1 0 0). On Li(1 1 0) (Figure 2 b), in general Li adsorption also becomes weaker for larger surface strain at all adsorption sites, only the short‐bridge position exhibits an opposite behavior for the compressed Li(1 1 0) surfaces. Na(1 1 0) (Figure 2 c) exhibits an uniform behavior on all Na adsorption sites, whereas Mg(0 0 0 1) (Figure 2 d) exhibits a nonuniform dependence of the Mg adsorption energies on the lattice strain at the bridge, hcp, and fcc sites, and the maximum binding energies are on the nonstrained or slightly expanded surfaces. Similar nonuniform behaviors were on the other considered surfaces. Overall, the changes in adsorption energy are relatively small, below 50 meV upon a lattice distortion of 1 %.

Notably, on late transition metals, typically, stronger binding on expanded surfaces has been observed.^23^, ^24^, ^25^ Within the d‐band model,^46^ this has been explained by a expansion‐induced upshift of the d‐band.^23^, ^47^ Interestingly, for early d‐band metals the opposite trend has been found,^48^ also in agreement with predictions of the d‐band model. Now except for Zn, which has a filled 3d band, no d‐band metals have been considered in this study, just metals with sp bands. Furthermore, for the 3d noble metal Cu no clear trends in adsorption energies on the most stable surface terminations as a function of lattice strain has been found.^49^ Hence again, in contrast to d‐band metals, apparently there is no simple model that can explain the dependence of adsorption energies on applied strain for sp metals.

### Diffusion barriers

We used the calculated adsorption energies to estimate the terrace diffusion barriers by Equation (5):

(5) $$E{}_{\mathrm{diff}}=E{}_{\mathrm{trans}}-E{}_{\min}$$

in which *E*_trans_ represents the energy of the transition state and *E*_min_ refers to the adsorption energy on the most favorable adsorption site. We emphasize that we only consider hopping diffusion processes on terraces in this work. So‐called exchange processes can be more favorable at step sites^12^, ^50^, ^51^, ^52^ but their explicit consideration is beyond the scope of the present study. Furthermore, Gaissmaier et al.^52^ showed that for adsorption particularly at the bridge positions of Li surfaces, the relaxation of more than two layers is necessary to get converged results. Again, this is beyond the scope of the present work. It might also be argued whether deep‐lying layers have the time to relax during the short time of the diffusion process.

**Figure 3:**
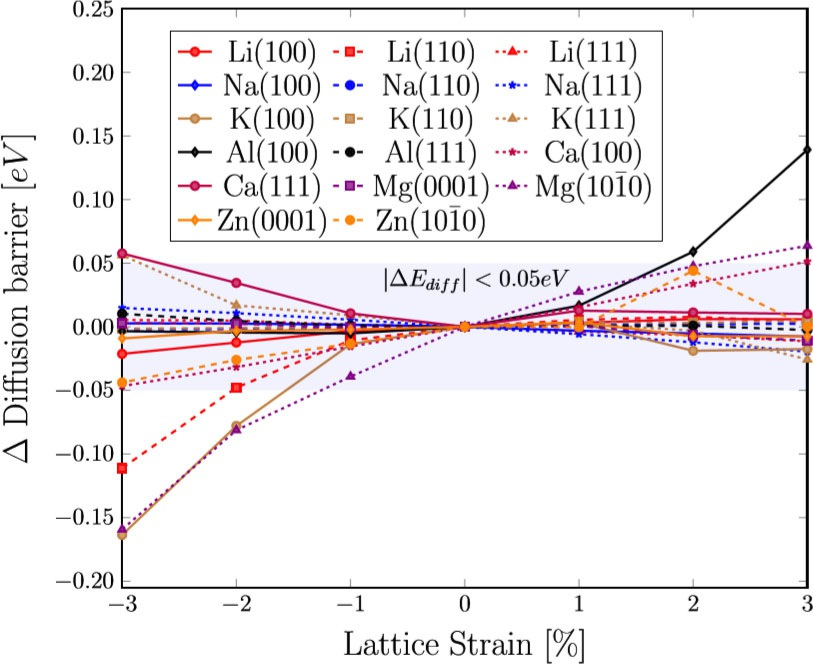
Change of the diffusion barrier along the minimum energy path with respect to lattice distortion for all investigated surfaces.

We summarize the change of the diffusion barriers as a function of the lattice strain in Figure 3. The variation in the height of the self‐diffusion barrier is indeed negligible for nearly all investigated systems, and the majority of the barriers vary by less than 50 meV, many of them by even below 20 meV. Only the Al(1 0 0) surface shows a significant increase in the diffusion barrier under expansion, whereas under compression, the K(1 0 0) and Mg(1 0 1 ¯0) surfaces display a decrease in their respective minimum energy path barriers. However, notably, the other considered Al, K, and Mg surfaces do not exhibit such a strong variation. In contrast to the effect on the surface and adsorption energies, the variation of the diffusion barrier does not correlate with the cohesive energy. Furthermore, the variation of the diffusion barriers with respect to surface strain does also not depend on the absolute height of the diffusion barriers, and it is of a similar order of magnitude for most surface terminations.

The weak dependence of diffusion barriers on lattice strain might come as a surprise if we consider that some adsorption energies show a strong dependence. However, one has to take into account that diffusion barriers correspond to the difference of the energies of the transition state and the initial adsorption state. Both of these energies are influenced by surface strain, but a similar dependence of both energies on the strain leads to a cancellation effect and results in a weak variation of the height of diffusion barriers with lattice strain. A similar phenomenon has been found for reaction barriers in methanol oxidation in heterogeneous catalysis on Cu(1 1 0).^53^ Although all binding energies of the reaction intermediates became larger upon lattice expansion in this system, the reaction barriers showed no clear trend as a function of lattice strain because of cancellation effects.

Although the lattice strain does not seem to affect the diffusion barriers along the minimum energy path between the most favorable adsorption positions strongly, it does affect a property of the potential energy surface (PES) that we call roughness. The roughness of the PES can be defined as the energy difference between the least and the most favorable adsorption site on the surface [Eq. (6)]:

(6) Erough=Emaxads-Eminads

**Figure 4:**
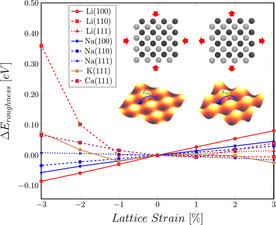
Change of the PES roughness of Li, Na, K, and Ca surfaces. Inset: representation of the change in PES roughness, preferred adsorption site in blue, transition state in red.

Notably, we did not explicitly sample the whole surface to find the energetically least favorable position, we selected the positions with the highest and lowest adsorption energy from our sampling of the high‐symmetry surface sites. The surface roughness can be of interest as it quantifies the maximum variation in the PES that is still relevant for the mobility on the surface. A very smooth PES would indicate high mobility, whereas a surface with a high PES roughness can be associated with slower diffusion. Such a distinction is not possible by only taking the primary diffusion process into account. The effect of strain on the PES roughness was more pronounced than the effect on the minimum diffusion barrier and also more monotonic. We observe a general trend in the PES roughness (Figure 4). For nearly all considered surfaces, the PES roughness increases almost linearly with surface expansion. The exceptions are Li(1 1 0), Ca(1 1 1), and K(1 1 1) for which the surface roughness increases under both expansion and compression. The trend of the decreased roughness of the PES under compression and the increased roughness under expansion can be understood by looking at the extreme cases. If the atoms in the lattice were to be compressed into an extremely tight formation, a distinction between the different surface positions might become impossible, whereas under extreme expansion, one would eventually end up with noninteracting atoms in a vacuum with a strong variation in the adsorption energy.

## Conclusions

We studied the influence of strain on several surface properties of metals that are used as charge carriers in batteries. The variation in the surface energies correlates with the cohesive energy of the respective metals. For almost all surfaces, the surface energy was decreased by strain effects. However, there is no universal preference for compression or expansion with regard to the decrease of the surface energy as it is dependent on both the surface termination and the element in question.

The strain dependence of the adsorption energy is even less universally predictable as it is dependent on the adsorption position, the surface termination, and the particular metal. No correlation of the strain dependence of the adsorption energies with the cohesive energy of the metal was found. Some sites even exhibit a completely disproportionate dependence on the strain compared to other sites of the same element and surface.

As a result of the lack of clear trends and rather strong variations in adsorption energy for specific sites, the strain effects in adsorption can apparently only be captured on a case‐by‐case basis. Strain‐related effects on the self‐diffusion barriers are rather small for nearly all of the metals and surfaces considered. This can be attributed to cancellation effects in the strain dependence of the initial and the transition states in diffusion. Only the overall roughness of the potential energy surfaces for adsorption exhibits a linear dependence on the strain for a number of metals. Therefore, it seems reasonable to conclude that the strain‐induced changes observed experimentally in the growth behavior of dendrites are not caused by changes in the atomic transport but rather because of the effect of strain on the morphology of the growing dendrites on a larger‐length scale and on the structural properties of electrode–electrolyte interfaces and solid–electrolyte interphases. To substantiate this, however, requires more detailed studies that also consider the electrochemical environment.

## Conflict of interest

*The authors declare no conflict of interest*.

## Supporting information

As a service to our authors and readers, this journal provides supporting information supplied by the authors. Such materials are peer reviewed and may be re‐organized for online delivery, but are not copy‐edited or typeset. Technical support issues arising from supporting information (other than missing files) should be addressed to the authors.

Supplementary
