# Supplementary material for: Strain Dependence of Metal Anode Surface Properties
Source: ChemSusChem. 2020 May 27;13(12):3147–53. doi: 10.1002/cssc.202000709 (PMC7318162; doi:10.1002/cssc.202000709)
Supplement: Supplementary file 1 — Supplementary [file CSSC-13-3147-s001.pdf]

Author Contributions

D.S. Investigation: Lead; Methodology: Equal; Visualization: Lead; Writing - Original Draft: Lead; Writing - Review & Editing: Supporting
A.G. Conceptualization: Lead; Funding acquisition: Lead; Methodology: Equal; Project administration: Lead; Supervision: Lead; Writing - Original Draft: Supporting; Writing - Review & Editing: Lead.
